# K336I mutant actin alters the structure of neighbouring protomers in filaments and reduces affinity for actin-binding proteins

**DOI:** 10.1038/s41598-019-41795-w

**Published:** 2019-03-29

**Authors:** Nobuhisa Umeki, Keitaro Shibata, Taro Q. P. Noguchi, Keiko Hirose, Yasushi Sako, Taro Q. P. Uyeda

**Affiliations:** 1 Cellular Informatics Lab., RIKEN, Wako, Saitama, 351-0198 Japan; 2 Biomedical Research Institute, National Institute of Advanced Industrial Science and Technology (AIST), Tsukuba, Ibaraki, 305-8562 Japan; 3 National Institute of Technology, Miyakonojo College, Miyakonojo, Miyazaki, 885-8567 Japan; 4 Department of Physics, Waseda University, Shinjuku, Tokyo, 169-8555 Japan; 5 Present Address: Advanced ICT Research Institute, National Institute of Information and Communications Technology (NICT), Kobe, Hyogo, 651-2492 Japan

## Abstract

Mutation of the Lys-336 residue of actin to Ile (K336I) or Asp (K336E) causes congenital myopathy. To understand the effect of this mutation on the function of actin filaments and gain insight into the mechanism of disease onset, we prepared and biochemically characterised K336I mutant actin from *Dictyostelium discoideum*. Subtilisin cleavage assays revealed that the structure of the DNase-I binding loop (D-loop) of monomeric K336I actin, which would face the adjacent actin-protomer in filaments, differed from that of wild type (WT) actin. Although K336I actin underwent normal salt-dependent reversible polymerisation and formed apparently normal filaments, interactions of K336I filaments with alpha-actinin, myosin II, and cofilin were disrupted. Furthermore, co-filaments of K336I and WT actins also exhibited abnormal interactions with cofilin, implying that K336I actin altered the structure of the neighbouring WT actin protomers such that interaction between cofilin and the WT actin protomers was prevented. We speculate that disruption of the interactions between co-filaments and actin-binding proteins is the primary reason why the K336I mutation induces muscle disease in a dominant fashion.

## Introduction

Actin is involved in many crucially important cellular functions such as muscle contraction, cytokinesis, cell motility, intracellular transport, adhesion, cell signalling, endocytosis, and exocytosis^1–3^. To date, a number of dominant negative actin mutations have been identified from genetic screens in model organisms and genetic analyses of human diseases^4–11^, and some of them were biochemically characterised^12–14^. In general, however, purification and biochemical analyses of recombinant dominant negative actins are difficult, particularly when the mutation dominantly interferes with essential functions of actin in expression host cells^6^. To address this problem, we previously developed a fusion protein of actin and thymosin-β^15^. The thymosin-β moiety prevents recombinant mutant actin from copolymerising with endogenous actin in host cells so that the mutant actin does not exhibit toxicity. After extraction and purification of the fusion protein from host cells, recombinant mutant actin is separated from the thymosin-β moiety by chymotryptic digestion^15^. We have used this expression system to biochemically characterise several dominant negative mutant actins^16–18^.

The Lys-336 residue of actin is located near the ATP binding site (Supplementary Fig. S1) and is conserved among all known actins. It has been reported that mutation of Lys-336 of alpha-actin to Ile or Glu in muscle cells causes congenital myopathy^8,9^. D’Amico *et al*. previously reported that dominant negative K336E actin, isolated from human muscle biopsy, impairs interactions between alpha-actinin and myosin II^19^. Their results also suggested that the weaker binding of K336E actin to alpha-actinin, which anchors actin filaments to the Z-line, leads to lower force generation and impaired force transmission in muscle cells, resulting in muscle disease. However, biochemical characterisation of the mutant actin was insufficient, probably due to the small amount of actin obtainable from human muscle biopsy. Using the thymosin-β expression system, we purified a *Dictyostelium discoideum* K336I mutant actin to further examine the effect of K336I mutation on the function of actin filaments at the molecular level. Our *in vitro* studies revealed that K336I actin forms apparently normal co-filaments with WT actin, but that interactions of co-filaments with alpha-actinin, cofilin, and myosin II are impaired. Most notably, K336I actin in co-filaments alters the structural properties of neighbouring WT actin protomers, and prevents them from interacting with cofilin.

## Results

### Expression and purification of recombinant K336I actin

K336I actin was expressed in *Dictyostelium* cells as a fusion protein with thymosin-β and a His-tag. After extraction and purification of the fusion protein, actin was separated from the thymosin-β and His-tag moieties by chymotryptic digestion, yielding a 42-kDa actin band when subjected to SDS-PAGE (Supplementary Fig. S2). We further purified the recombinant K336I actin by Q-Sepharose column chromatography followed by a cycle of polymerisation and depolymerisation (Supplementary Fig. S2). The average yield of K336I actin was approximately 5 mg from 6.0 litres of culture (6.0 × 10^6^ cells/litre), comparable to WT actin preparations.

### Properties of K336I mutant globular actin (G-actin)

**Figure 1:**
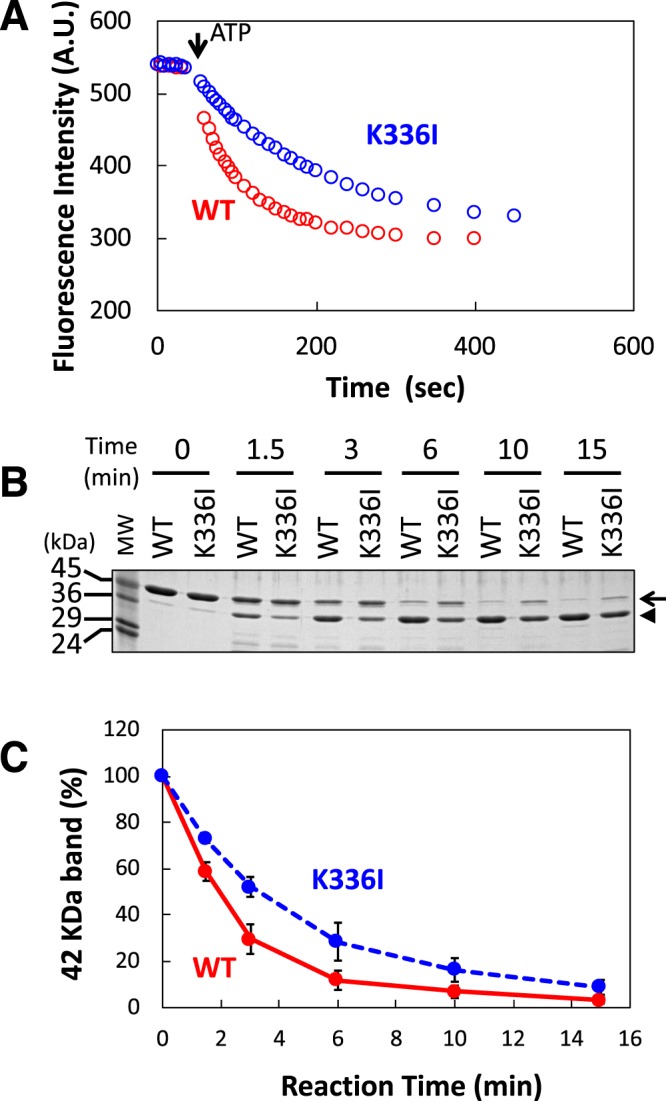
Properties of purified K336I monomeric actin. (**A**) The release rate of ε-ATP from K336I and WT actin monomers was monitored by measuring the decrease in fluorescence intensity after the addition of regular ATP (1 mM) to 20 µM K336I or WT G-actin that had been incubated with ε-ATP. The dissociation rates of ε-ATP from K336I mutant actin and WT actin were 0.009 ± 0.001 s^−1^ and 0.025 ± 0.005 s^−1^, respectively (mean ± SE, *N* = 3). There is a statistically significant difference between K336I and WT actin (*t*-test, *p* < 0.01). (**B**) K336I and WT actin (4 µM) were treated with 0.5 µg/ml subtilisin for the indicated time, and analysed by SDS-PAGE. Arrows indicate intact actin bands (42 kDa) and arrowheads indicate digested actin bands (36 kDa). MW, molecular weight markers. Full-length gel image is shown in Supplementary Fig. S3. (**C**) Quantitation of intact actin bands from Coomassie-stained gels, including the one shown in (**B**). After 3 minutes of subtilisin digestion, 52.1 ± 4.3% of K336I G-actin was intact, vs. 29.6 ± 6.4% of WT G-actin (mean ± SE, *N* = 3).

We hypothesised that mutation of Lys-336 to Ile would affect the conformation of the ATP binding site because the side chain of Lys-336 indirectly contacts the nucleotide in the ATP binding pocket through a water molecule^20^ (Supplementary Fig. S1). To test this, we measured the nucleotide exchange rate of K336I mutant G-actin using a fluorescent ATP analogue, ε-ATP, and compared it to that of WT G-actin (Fig. 1A). The release rate of ε-ATP from K336I G-actin was lower than from WT G-actin (Fig. 1A). This result suggests that conformation of the ATP binding site of K336I G-actin differs from that of WT G-actin.

It is well known that the conformation of the ATP binding site influences the structure of D-loop (residues 39–51) in subdomain 2^21,22^, which faces the adjacent actin subunit in filamentous actin (F-actin) and plays an important role in polymerisation^23^ (Supplementary Fig. S1). Previous reports showed that the cleavage rate of the D-loop by the serine protease subtilisin depends on its structure^12,17,24^. Therefore, we employed a subtilisin digestion assay to investigate the conformation of the D-loop of K336I G-actin. Consistent with previous reports^25–27^, subtilisin cleavage of G-actin, presumably between Met47-Gly48^12,27^, yielded a 36 kDa fragment (Fig. 1B,C and Supplementary Fig. S3). Densitometric analysis revealed that K336I G-actin was less susceptible to subtilisin protease than WT G-actin (Fig. 1C), suggesting that mutation of Lys-336 to Ile also affects the conformation of D-loop.

### Polymerisation and depolymerisation of K336I actin

Because the structure of the D-loop of K336I G-actin differed from that of WT actin, we next investigated the effects of K336I mutation on polymerisation and depolymerisation. K336I actin was induced to polymerise and depolymerise by the addition of salt and Latrunculin A, respectively, and we observed changes in light scatter (Supplementary Fig. S4). Unexpectedly, changes in light scatter were similar between K336I and WT actin. Moreover, the critical concentration of K336I actin, estimated by the ultracentrifugation method, was 0.72 µM, which was comparable to that of WT actin (0.58 µM) (Supplementary Fig. S5). These results suggest that mutation of the Lys-336 residue does not significantly affects actin’s abilities to polymerise and depolymerise. Consistent with this, K336I actin filaments appeared normal when observed by electron microscopy (Supplementary Fig. S6) and fluorescence microscopy (Supplementary Fig. S7).

In patients with congenital myopathy who are heterozygous at the alpha-actin locus, K336I actin is likely to be present in muscle cells at the same concentration as WT actin. Thus, we next examined whether K336I actin formed co-filaments with WT actin when the two proteins were mixed in G-buffer and induced to polymerise by the addition of salt. Fluorescence microscopy of Alexa Fluor–labelled actins showed that K336I actin can form co-filaments with WT actin (Supplementary Fig. S7).

### Interactions of K336I actin filaments with alpha-actinin and myosin

**Figure 2:**
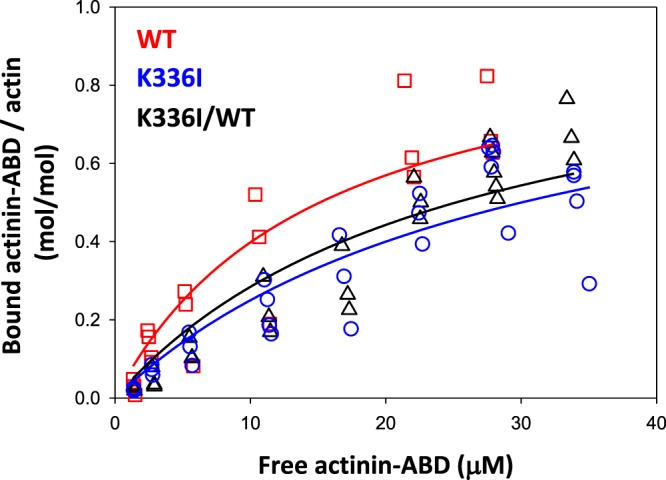
Co-sedimentation of K336I actin filaments and alpha-actinin ABD. Solid lines show data fit using the following equation: [alpha-actinin ABD]_bound_/[F-actin] = [alpha-actinin ABD]_free_/K_d_ + [alpha-actinin ABD]_free_. The dissociation constants (K_d_) of alpha-actinin ABD from actin filaments were as follows: K336I homo-filaments (*blue circles*), 30.0 ± 2.7 µM; WT homo-filaments (*red squares*), 15.1 ± 2.0 µM; and K336I/WT co-filaments (*black triangles*), 25.2 ± 2.2 µM (mean ± SE, *N* = 4). There is a statistically significant difference between WT homo-filaments and K336I homo-filaments (*t*-test, *p* < 0.001) as well as between WT homo-filaments and K336I/WT co-filaments (*t*-test, *p* < 0.01).

A previous semi-quantitative study demonstrated that interactions of actin filaments with alpha-actinin and myosin II were significantly attenuated by K336E mutation^19^. We examined the binding affinity of K336I actin and the actin-binding domain (ABD) of *Dictyostelium* alpha-actinin using quantitative co-sedimentation assays and found that the K336I mutation decreased the affinity between actin filaments and the alpha-actinin ABD. Binding affinity was decreased by 50% for K336I homo-filaments and 16% for K3336I/WT co-filaments as compared to WT homo-filaments (Fig. 2).

**Figure 3:**
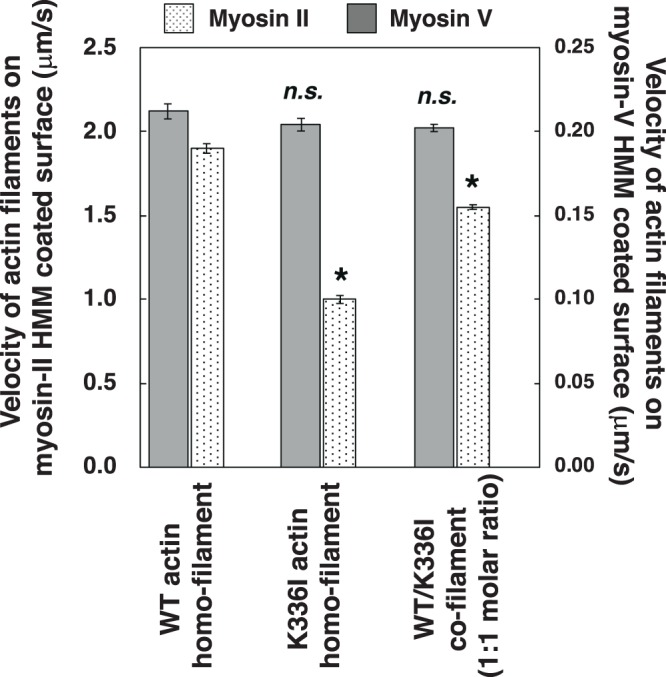
Sliding velocities of K336I and WT actin filaments on myosin II-HMM and myosin V-HMM. Velocities (mean ± SE) on surfaces of myosin II-HMM were as follows: K336I homo-filaments, 1.00 ± 0.02 µm/s (*N* = 137); WT homo-filaments, 1.90 ± 0.03 µm/s (*N* = 110); and K336I/WT co-filaments, 1.55 ± 0.03 µm/s (*N* = 165). Velocities on surfaces of myosin V-HMM were as follows: K336I homo-filaments, 0.204 ± 0.003 µm/s (*N* = 40); WT homo-filaments, 0.212 ± 0.004 µm/s (*N* = 40); and K336I/WT co-filaments, 0.202 ± 0.002 µm/s (*N* = 40). Asterisks indicate a statistically significant difference compared to WT homo-filaments (*t*-test, *p* < 0.001). Statistically insignificant differences are indicated by “*n.s*.”.

We also performed *in vitro* motility assays to assess the interaction of K336I actin filaments with myosin. The velocities of K336I actin homo-filaments and K336I/WT co-filaments on surfaces coated with rabbit skeletal muscle myosin II heavy meromyosin (HMM) were decreased by approximately 47% and 18%, respectively, compared to that of WT homo-filaments (Fig. 3). These results indicate that the K336I mutation significantly disturbs the interaction with myosin II as well. Interestingly, when we repeated the assay using human myosin V, motility was unaffected, suggesting that disruption of the actin–myosin interaction by the K336I mutation is myosin class-specific (Fig. 3).

### Effect of K336I mutation on cofilin binding

**Figure 4:**
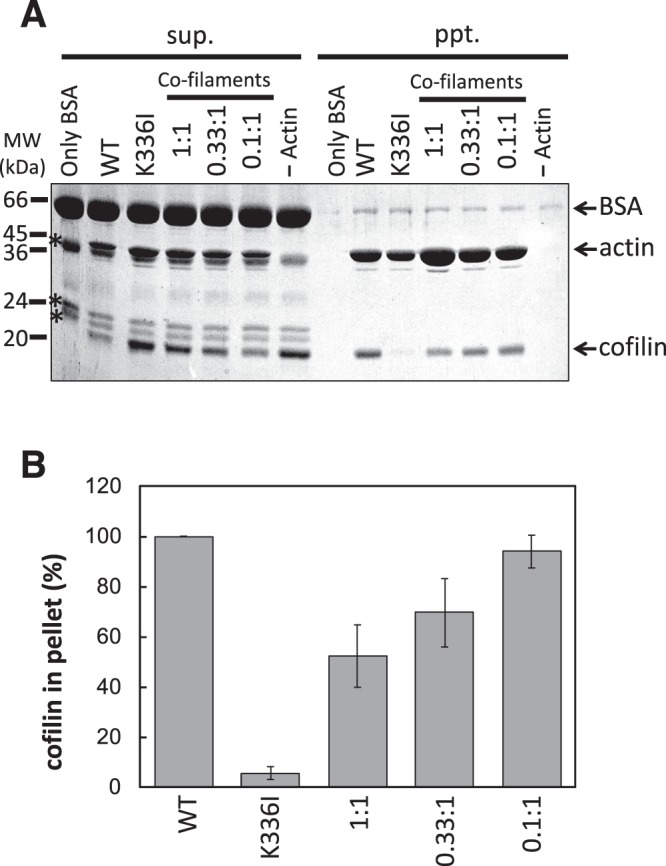
Cofilin binding. (**A**) Co-sedimentation of 2.5 µM cofilin with 5 µM K336I homo-filaments, 5 µM WT homo-filaments, and K336I/WT co-filaments consisting of 5 µM WT actin and 5 (1:1), 1.7 (0.33:1), or 0.5 µM (0.1:1) K336I mutant actin was analysed by SDS-PAGE. sup, supernatant; ppt, pellet after ultracentrifugation. Asterisks indicate contaminating proteins present in the BSA solution. Full-length gel image is shown in Supplementary Fig. S8. (**B**) Densitometric analysis of three Coomassie-stained SDS-gels, including the one shown in (**A**), determined that 5.6 ± 2.6%, 52.2 ± 12.4%, 69.5 ± 13.6%, and 93.9 ± 6.5% (mean ± SE) of cofilin co-sedimented with K336I homo-filaments, 1:1, 0.33:1, and 0.1:1 co-filaments, respectively. The amount of cofilin in each pellet was normalized to the amount in the pellet of WT homo-filaments.

Cofilin is a major actin filament–severing protein^28,29^, but the dominant negative actin mutations D11Q/N and G146V prevent interaction with cofilin^5,17^. We examined the effect of K336I mutation on cofilin binding using a co-sedimentation assay, which was performed under slightly acidic conditions to suppress actin-severing activity^30^. Although cofilin efficiently bound to 5 µM WT homo-filaments under our experimental conditions, cofilin bound only very weakly to 5 µM K336I homo-filaments (Fig. 4 and Supplementary Fig. S8). Interestingly, when 5 µM WT actin was copolymerised with increasing concentrations (0.5, 1.7, and 5.0 µM, resulting in the molar ratios of K336I:WT actin = 0.1:1, 0.33:1, and 1:1, respectively, as shown in Fig. 4) of K336I actin, the amount of bound cofilin decreased progressively, indicating that cofilin bound to WT protomers in the co-filaments less efficiently than to those in WT homo-filaments. The results suggest that K336I protomers in the co-filaments affect the structure of neighbouring WT protomers and reduce their affinity for cofilin.

**Figure 5:**
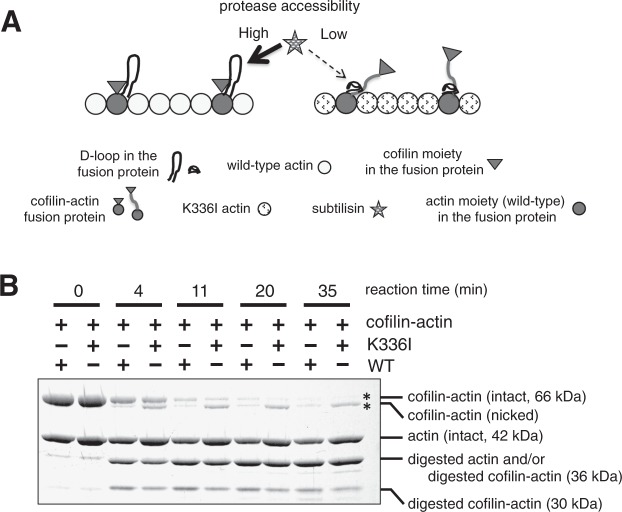
Copolymerisation of cofilin–actin fusion protein with K336I actin affects cleavage of the D-loop in the fusion protein. As shown in our previous report^31^, the fusion protein polymerises and depolymerises normally in a salt-dependent manner, and the cofilin moiety in the fusion protein has a pH-sensitive depolymerisation and severing activities of actin filaments. These functional properties of the cofilin moiety are similar to that of normal cofilin. Thus, there is seems to be no steric problem between the actin and cofilin moieties in the fusion protein in terms of interaction with other actin molecules. (**A**) Schematic drawing showing the detection of structural changes of the actin moiety in the fusion protein when copolymerised with K336I actin. In a co-filament, when K336I actin protomers affect the structure of the WT actin moiety of the fusion protein, they reduce the affinity of that actin moiety for cofilin moiety, causing its D-loop to have reduced susceptibility to subtilisin cleavage. The D-loop of actin (except for the actin moiety in the fusion protein) is not shown. (**B**) Subtilisin digestions of K336I/cofilin–actin co-filaments or WT/cofilin–actin co-filaments. Cofilin–actin fusion protein with an intact D-loop became slightly smaller after treatment with subtilisin, presumably due to nicking in the cofilin moiety by subtilisin, as shown in our previous report^31^. Digestion of the D-loop in the cofilin–actin fusion protein yielded bands of approximately 30 and 36 kDa, and did not yield a 42 kDa band^31^. The amount of undigested D-loop of the fusion protein (the sum of the 66 kDa band and the slightly smaller nicked band, marked by asterisks) in the K336I/cofilin–actin co-filaments was greater than in the WT/cofilin–actin co-filaments when digested for the same length of time. Nicking of the cofilin moiety in the fusion protein was accelerated by copolymerisation with K336I actin, and was most evident after 11 minutes of subtilisin digestion. This is consistent with the results of co-sedimentation experiments (Fig. 4), and supports the model shown in (**A**). Full-length gel image is shown in Supplementary Fig. S8.

Recently, we reported that a cofilin–actin fusion protein is a useful tool for investigating cooperative conformational changes of actin filaments^31^. By copolymerising this fusion protein with K336I actin, we were able to further investigate the effect of K336I on neighbouring actin protomers. It is known that the D-loop of actin in filaments is slowly cleaved by subtilisin, but when cofilin is bound, the cleavage reaction is significantly accelerated^24^. Consistent with our previous report^31^, in co-filaments of WT actin and cofilin–actin fusion protein, the D-loop of the fusion protein was rapidly digested by subtilisin (Fig. 5B and Supplementary Fig. S8). By contrast, when the fusion protein copolymerised with K336I actin, the cleavage rate of the D-loop in the fusion protein significantly decreased. This is consistent with the result of Fig. 4 and supports the conclusion that mutation of Lys-336 to Ile affects the structure of neighbouring actin protomers in the filament and thereby reduces the affinity for cofilin.

### Pi release from K336I mutant actin

As mentioned above, cofilin does not bind K336I actin filaments well. Cofilin preferentially binds to ADP-actin protomers in filaments; thus, this lack of binding might be due to inhibition of ATP hydrolysis or Pi release following ATP hydrolysis^32^. We measured the ATPase activity of actin during polymerisation and found that K336I actin homo-filaments have the ability to hydrolyse ATP and release Pi during polymerisation, and have slightly accelerated ATP hydrolysis activity compared to WT actin (Supplementary Fig. S9). This result suggests that inhibition of cofilin binding is not due to defects in ATP hydrolysis or Pi release.

## Discussion

Although the K336E/I mutation of alpha-actin has been shown to be dominant negative in humans^8,9^, the effect of this mutation on the function of actin filaments at the molecular level has not been elucidated. Our *in vitro* studies using recombinant K336I actin revealed that although K336I actin forms co-filaments with WT actin (Supplementary Fig. S7), interactions of K336I/WT co-filaments with alpha-actinin (Fig. 2), myosin II (Fig. 3), and cofilin (Fig. 4) were impaired. Most importantly, a structural change in K336I actin protomers affects neighbouring WT actin protomers in K336I/WT co-filaments (Fig. 5) and allosterically reduces their affinity for cofilin (Fig. 4).

The function and proper arrangement of actin filaments are critical for muscle contraction, and depend on interactions with a number of actin-binding proteins. For example, interactions of actin filaments with alpha-actinin, myosin II, and cofilin are required for achieving the assembly and maintenance of muscle fibres^33^, force-production^34^, and the turnover of sarcomeric actin^35^, respectively. Therefore, it is plausible that disruption of the interactions between these proteins and actin filaments induces muscle disease^36–38^.

Previous molecular dynamics studies suggested that the Lys-336 residue and several residues on the D-loop are involved in interactions with cofilin and myosin II^39,40^. Consistent with this, our studies showed that the K336I mutation impairs interactions with those proteins (Figs 3 and 4). However, this does not necessarily mean that the phenotype of affected muscle cells is solely caused by disruption of the binding site by substitution of the side chain of Lys-336. It is possible that the binding of an actin-binding protein induces a conformational change in an actin protomer and this conformational change may be propagated along the filament, recruiting additional actin-binding protein to neighbouring protomers^41–46^. Such cooperative binding may be important for achieving the cellular functions of actin^44^. For example, cofilin alters the twist of actin filaments, and this conformational change propagates to neighbouring actin protomers in the same filament^31,43,45,47–49^. This cooperative conformational change induces further binding of cofilin molecules that results in cooperative binding, and this may contribute to the severing function of cofilin^31,45,50^. Our data clearly indicated that K336I actin impairs the interaction of cofilin with WT actin protomers in K336I/WT co-filaments (Fig. 4), implying that K336I actin protomers inhibit cofilin-induced cooperative conformational changes of the co-filaments, resulting in defective cooperative cofilin binding.

Conformational freedom of the hinge region connecting the large and small domains of actin might be important for the conformational change of actin filaments and required for the binding of certain proteins^51^. We previously reported that the G146V mutation, which is positioned in the hinge region (Supplementary Fig. S1) and is dominant lethal in yeast, also inhibits cooperative binding of cofilin^5^. Furthermore, motility and force generation of G146V actin filaments with myosin II are strongly impaired *in vitro*, even though motility and force generation with myosin V are normal^5,18^. These phenotypes of G146V actin are qualitatively very similar to those of K336I actin. Since the G146V mutation likely perturbs the structural change between the large and small domains of actin^52^, it is plausible that the interaction of G146V actin with cofilin and myosin II is impaired despite the fact that cofilin and myosin II do not interact directly with Gly-146. Because Lys-336 is also in the hinge region^51^ (Supplementary Fig. S1) and the biochemical properties of K336I actin are similar to those of G146V actin, we speculate that K336I mutation also perturbs the structural change between the large and small domains.

Of particular interest is the fact that both G146V and K336I mutant actins show impaired motility with myosin II but not with myosin V, implying that the structural requirements of actin filaments are different for fast, non-processive myosin II and slow, processive myosin V motilities. These two mutations may impair structural requirements for myosin II through a common mechanism. Further studies are needed to understand the inhibitory mechanism, with the ultimate goal of understanding the conformational requirements of actin for productive interaction with myosin II.

The side-chain of Lys-336 indirectly contacts the nucleotide in the ATP binding pocket through a water molecule (Supplementary Fig. S1). Our nucleotide exchange assay showed that the mutation of Lys-336 to Ile increased the nucleotide-binding capability of actin (Fig. 1A), which is difficult to explain by disruption of the ATP binding interface. One possible explanation is that impairment of the conformational change between the large and small domains of actin by the K336I mutation indirectly affects the nucleotide-binding ability. We speculate that the abnormal conformational change induced by the mutation, rather than slower nucleotide exchange, causes impaired cellular function of K336I actin.

In summary, our *in vitro* studies demonstrated that K336I mutant actin is able to form co-filaments with WT actin and disrupts interactions of these co-filaments with alpha-actinin, cofilin, and myosin II. K336I actin protomers in co-filaments alter the structure of neighbouring WT actin protomers and thereby allosterically and cooperatively reduce their affinity for cofilin.

## Methods

### Plasmid construction

pTIKL ART^15^ contains an ART gene, which is the *Dictyostelium act15* gene modified to carry four unique restriction sites (the AR gene), followed by a Gly-based linker, a synthetic human thymosin-β gene, and a His-tag. The K336I mutation was synthesised using a PCR-based method and subcloned into pTIKL ART after confirmation by DNA sequencing. The mutated sequence is CCACCAGAACGTATTTACTCTGTCTGGA, with mutated nucleotides underlined. *Dictyostelium* alpha-actinin ABD (amino acids 6–250) cDNA was cloned between KpnI and PstI sites of the pCold I vector (Takara Bio) containing a TEV protease cleavage sequence between NdeI and KpnI sites^45^.

### Preparation of proteins

Recombinant WT and K336I actins were expressed and purified as described previously^16^. Briefly, Ax2 or KAx3 *Dictyostelium discoideum* cells were transfected by electroporation with the pTIKL-based plasmids described above and grown in HL5 medium containing 40 µg/mL G418. The cells were harvested, washed, resuspended, and disrupted in the extraction buffer (20 mM HEPES pH7.4, 0.5 M NaCl, 2 mM MgCl_2_, 1 mM ATP, 7 mM β-mercaptoethanol, 5–10 mM imidazole pH 7.4, 0.25% Triton X-100, and protease inhibitors). After the cell lysates were centrifuged at 36,000 × *g* for 30 minutes at 4 °C, each ART in the supernatants was enriched using a Ni^2+^-NTA affinity column (Qiagen). The crude ARTs were dialysed against G-buffer (2 mM HEPES pH 7.4, 0.2 mM CaCl_2_, 0.1 mM ATP, and 0.5 mM dithiothreitol (DTT)), and then digested with chymotrypsin. The digested proteins were further purified by Q-Sepharose column chromatography (GE Healthcare).

The purification of *Dictyostelium* cofilin and cofilin–actin fusion protein was performed as described previously^31^. In brief, KAx3 wild type *Dictyostelium* cells were transfected with pTIKL cofilin–AR and grown in HL5 medium containing 40 μg/mL G418. After the cells were harvested and disrupted, cofilin–actin was purified from cell lysate using a Ni^2+^-NTA affinity column as described above. For further purification, the cofilin–actin solution was loaded onto an anti-FLAG M2 affinity agarose gel column (Wako Chemicals). After the column was washed, cofilin–actin was eluted with 0.1 mg/ml FLAG peptide dissolved in buffer (0.4 M NaCl, 10 mM HEPES pH 7.4, 1 mM MgCl_2_, 0.5 mM ATP, and 7 mM β-mercaptoethanol) and was dialysed against G-buffer containing 10% sucrose. The recombinant *Dictyostelium* cofilin was expressed in *Escherichia coli* Rosetta cells and purified using a Ni^2+^-NTA affinity column (Qiagen).

Rabbit skeletal myosin II-HMM was prepared by limited digestion^53^, and recombinant truncated human myosin V (myosin V-HMM) was purified as described previously^18^. Briefly, SF9 cells were coinfected with two separate viruses expressing the myosin V-HMM and calmodulin and were cultured at 28 °C. After 3 days, the cells were harvested and disrupted by sonication in buffer (0.3 M KCl, 20 mM HEPES pH 7.5, 1 mM MgCl_2_, 10 mM EGTA, 0.1 mg/mL calmodulin, 7 mM β-mercaptoethanol, 2 mM ATP, and protease inhibitors). The cell lysate was ultracentrifuged, and then the resultant supernatant was loaded onto an anti-FLAG M2 affinity agarose gel column. After the column was washed, human myosin V-HMM bound with calmodulin was eluted with 0.15 mg/ml FLAG peptide dissolved in buffer (0.3 M KCl, 10 mM HEPES pH 7.5, 1 mM MgCl_2_, 0.5 mM EGTA, and 7 mM β-mercaptoethanol).

The recombinant alpha-actinin ABD was expressed in *Escherichia coli* Rosetta cells and purified using a Ni^2+^-NTA affinity column (Qiagen). To remove the His-tag, the purified protein was treated with His-tagged TEV protease and passed through a Ni^2+^-NTA resin. Aliquots of these proteins were snap-frozen in liquid nitrogen and stored at −80 °C. The concentration of actin was determined by measuring absorption at 290 nm^54^ and the concentrations of other proteins were estimated using the Advanced Protein Assay (Cytoskeleton) with BSA as the standard.

### Nucleotide exchange assay

The nucleotide exchange assay was performed as described previously^55^ with some modifications. Unbound ATP was removed from 20 µM G-actin solution by ion exchange resin (Dowex, 1 × 80, 100–200 mesh) and equilibrated in buffer A (2 mM Tris-HCl pH 7.4, 0.2 mM CaCl_2_, and 0.2 mM DTT), then the actin was incubated with 0.2 mM 1, *N*^6^-ethenoadenosine 5′-triphosphate (ε-ATP; Sigma-Aldrich) overnight on ice. Unbound ε-ATP was removed by Dowex resin in buffer A, and ε-ATP–bound G-actin was diluted to 0.5 µM with assay buffer (2 mM HEPES pH 7.4, 0.2 mM CaCl_2_, and 1 mM DTT) just before measurement. The nucleotide exchange reaction was induced by addition of 1 mM unlabelled ATP and was measured at 22 °C using a fluorescence spectrophotometer (RF-5300PC; Shimadzu) with an excitation wavelength of 340 nm and emission wavelength of 410 nm.

### Subtilisin digestion assay

Subtilisin digestion assays were performed as described previously^31^. Briefly, homo-filaments and co-filaments of WT actin, K336I actin, and cofilin–actin were prepared by incubation of the appropriate mixture in buffer (50 mM KCl, 2 mM HEPES pH 7.4, 2.5 mM MgCl_2_, 0.5 mM EGTA, 0.2 mM ATP, and 0.5 mM DTT) at 22 °C for 30 minutes, then 500 mM PIPES pH 6.5 was added to achieve a final concentration of 20 mM. After a 30-minute incubation, the resultant actin filaments were digested by 5 µg/ml subtilisin (Sigma-Aldrich) at 25 °C. The reactions were terminated by addition of 1 mM phenylmethylsulfonyl fluoride, and the samples were analysed by SDS-PAGE. Densitometric analysis was performed using ImageJ version 1.46 software (National Institutes of Health). For G-actin digestion, WT or K336I actin (4 µM) in G-buffer (2 mM HEPES pH 7.4, 0.2 mM CaCl_2_, 0.1 mM ATP, 0.5 mM DTT) was treated with 0.5 µg/ml subtilisin at 25 °C. The reaction was stopped and analysed as described above.

### Polymerisation and depolymerisation assay

Polymerisation of WT or K336I actin (10 µM) was induced by addition of concentrated F-buffer and the resultant increase in light scatter was monitored at 360 nm at 22 °C using a fluorescence spectrophotometer. The final concentration of each component was 100 mM KCl, 2 mM HEPES pH 7.4, 2.5 mM MgCl_2_, 0.5 mM EGTA, 0.5 mM ATP, and 0.5 mM DTT. Depolymerisation of WT (5 µM) or K336I (5 µM) actin filaments in buffer (100 mM KCl, 10 mM HEPES pH 7.4, 2.5 mM MgCl_2_, 0.5 mM EGTA, 0.1 mM ATP, and 1 mM DTT) was induced by addition of 36.5 µM Latrunculin A (Wako Chemicals). The critical concentration of actin was determined by polymerising various concentrations of actin (0.5, 1, 1.5, 3, 5, and 10 μM) in precipitation buffer (2 mM Tris-HCl, pH 8.0, 0.2 mM CaCl_2_, 50 mM KCl, 1 mM MgCl_2_, 1 mM DTT, and 0.2 mM ATP) for 20 minutes, followed by separation of the resultant F- and G-actin by centrifugation at 300,000 × *g* for 15 minutes at 4 °C. The concentration of each form was determined by quantitative densitometry of Coomassie-blue–stained SDS-PAGE gels.

### Electron microscopy

WT or K336I actin filaments in EM buffer (10 mM potassium phosphate buffer pH 7.4, 25 mM KCl, 2.5 mM MgCl_2_, 0.2 mM ATP, and 0.5 mM DTT) were placed on carbon-coated copper grids and stained with 1% uranyl acetate. Actin filaments were observed using an FEI Tecnai F-20 electron microscope.

### Fluorescence microscopy

WT and K336I actins were labelled with Alexa Fluor 488 or Alexa Fluor 594 succinimidyl ester (Invitrogen) as described previously^17^. Homo-filaments and co-filaments were prepared by polymerising fluorescently labelled WT G-actin (4 µM), K336I G-actin (4 µM), or a 1:1 mixture of WT (2 µM) and K336I (2 µM) G-actins in buffer (100 mM KCl, 2 mM HEPES pH 7.4, 2.5 mM MgCl_2_, 0.5 mM EGTA, 0.4 mM ATP, 0.5 mM DTT) at 22 °C for 2 hours. The resultant actin filaments were observed using a fluorescence microscope (BX60; Olympus) equipped with an EM-CCD camera (C7190; Hamamatsu Photonics) at 25 °C.

### Alpha-actinin binding

Three types of F-actin, WT homo-filaments (3 µM), K336I homo-filaments (3 µM), and K336I/WT co-filaments (3 µM, WT: K336I = 1:1), were incubated with various concentrations of the alpha-actinin ABD in precipitation buffer for 20 minutes, and mixtures were then centrifuged at 300,000 × *g* for 15 minutes at 4 °C. The resultant supernatants (unbound alpha-actinin ABD) and pellets (F-actin and bound alpha-actinin ABD) were subjected to SDS-PAGE. Protein concentrations were determined as described above.

### *In vitro* motility assay

The *in vitro* motility assay was performed as described previously^18^. Briefly, rabbit skeletal muscle myosin II-HMM or recombinant human myosin V-HMM was introduced into a flow chamber with a nitrocellulose-coated surface. Rhodamine phalloidin–stabilised actin filaments were subsequently added to the chamber, and gliding of the filaments was initiated by adding ATP solution (25 mM KCl, 10 mM HEPES pH 7.4, 4 mM MgCl_2_, 1 mM EGTA, 1 mg/ml BSA, 10 mM DTT, 1 mM ATP, 200 μg/ml glucose oxidase, 30 μg/ml catalase, and 3 mg/ml glucose). The gliding velocity of each actin filament was determined using ImageJ software (National Institutes of Health).

### Cofilin binding

WT actin (5 μM), K336I actin (5 μM), and mixtures of WT actin (5 μM) with various concentrations of K336I actin were polymerised in buffer (50 mM KCl, 2 mM Tris-HCl pH 7.4, 2.5 mM MgCl_2_, 0.5 mM EGTA, 0.2 mM ATP, and 0.2 mM DTT) containing 1 mg/ml BSA for 2 hours at 22 °C, then 500 mM PIPES pH 6.5 and cofilin were added at a final concentration of 20 mM and 2.5 μM, respectively. After a 10-minute incubation, the mixtures were centrifuged at 300,000 × *g* for 10 minutes at 22 °C. The supernatant and pellet fractions were subjected to SDS-PAGE.

### Phosphate release assay

The time course of Pi release from polymerising actin was measured using an EnzChek Phosphate Assay Kit (Invitrogen). Actin (10 μM) was polymerised as described above in the presence of 2-amino-6-mercapto-7-methylpurine ribose and 1 unit/ml purine nucleotide phosphorylase, and the absorbance at 360 nm was monitored.

## Supplementary information

Supplementary Figures
