# Supplementary material for: K336I mutant actin alters the structure of neighbouring protomers in filaments and reduces affinity for actin-binding proteins
Source: Sci Rep. 2019 Mar 29;9:5353. doi: 10.1038/s41598-019-41795-w (PMC6441083; doi:10.1038/s41598-019-41795-w)
Supplement: Supplementary file 1 — Supplementary Figures [file 41598_2019_41795_MOESM1_ESM.pdf]

Supplementary Information

K336I mutant actin alters the structure of neighbouring protomers in filaments and reduces affinity for actin-binding proteins

Nobuhisa Umeki*, Keitaro Shibata, Taro Q.P. Noguchi,
Keiko Hirose, Yasushi Sako, and Taro Q.P. Uyeda

∗ corresponding author: nobuhisa.umeki@riken.jp

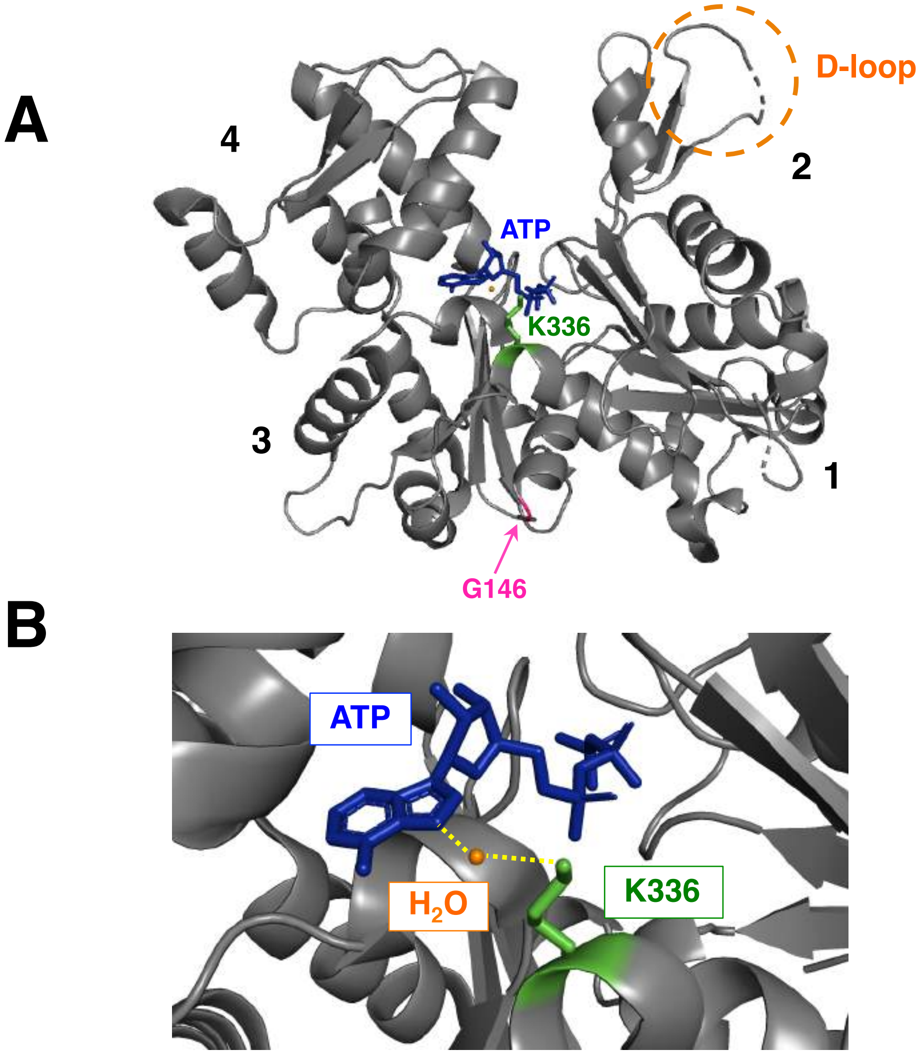

Supplementary Figure S1. Location of the Lys-336 residue. (A) Overall structure of ATP-bound G-actin (Protein Data Bank code, 1C0F). Lys-336 (green sticks) is located in the nucleotide-binding site. ATP and Gly-146 are indicated by blue and pink sticks, respectively. Numbers show the subdomains. The small domain comprises subdomains 1 and 2, and the large domain comprises subdomains 3 and 4. The D-loop, indicated by a dotted orange circle, is located in subdomain 2. (B) Enlarged view of the nucleotide-binding site of ATP-bound actin. The side chain of Lys-336 indirectly contacts ATP through a water molecule (orange dot). Broken yellow lines correspond to hydrogen bonds.

A

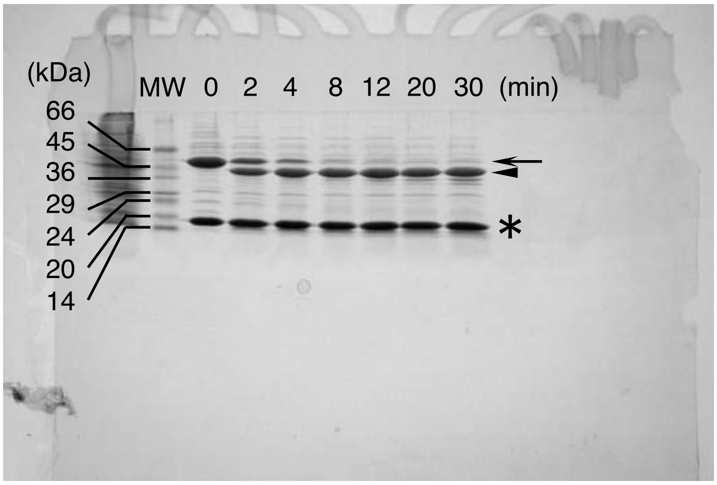

B

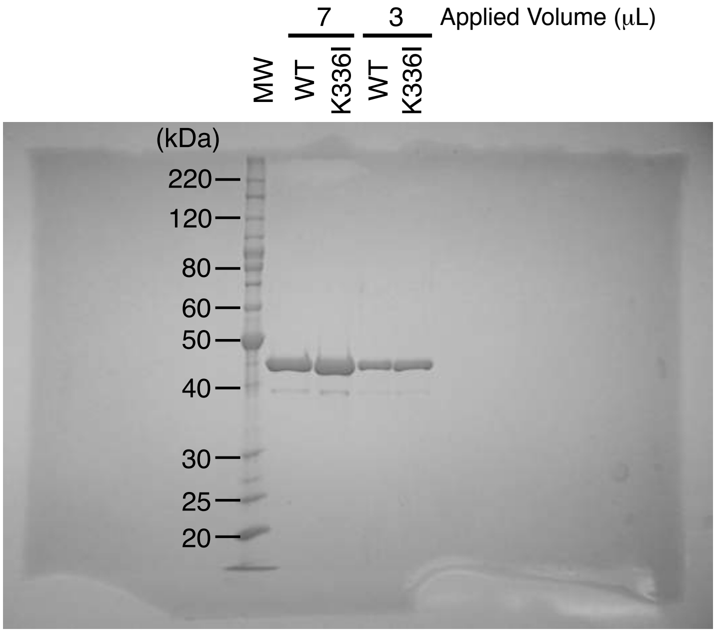

Supplementary Figure S2. Separation of K336I actin and thymosin-His tag moieties by chymotryptic digestion. (A) Crudely purified K336I–thymosin–His fusion protein was digested with 13.3 μg/ml chymotrypsin at 25 °C. Digestion was stopped at the indicated times. Arrow indicates undigested actin–thymosin–His fusion protein (51 kDa) and arrowhead indicates intact actin (42 kDa). Asterisk indicates an endogenous *Dictyostelium* His–containing protein, which was removed by subsequent anion-exchange chromatography. (B) Final products of K336I and WT actins. MW, molecular weight markers.

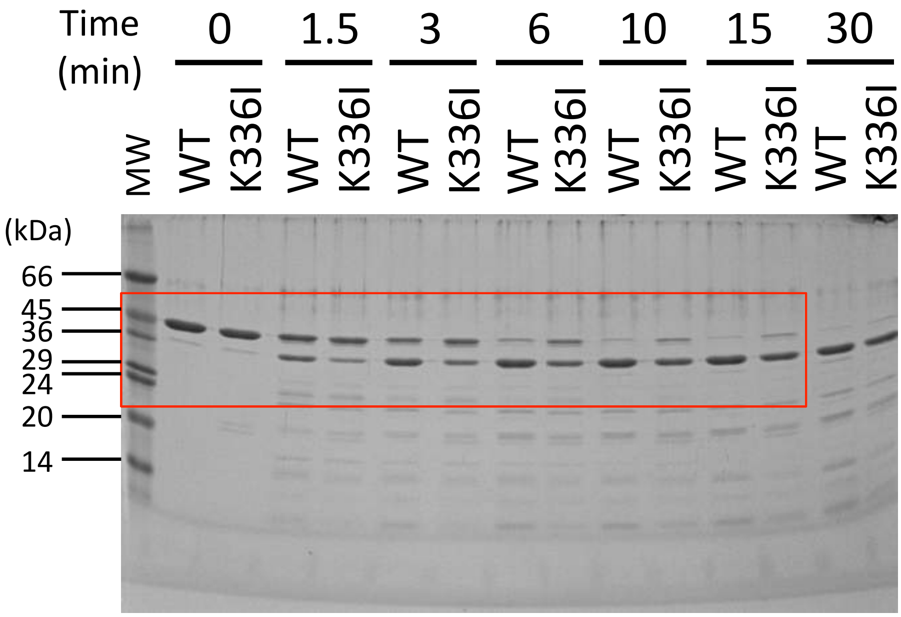

Supplementary Figure S3. Complete SDS-PAGE gel shown in Fig. 1B. The boxed area is shown in Fig. 1B. MW, molecular weight markers.

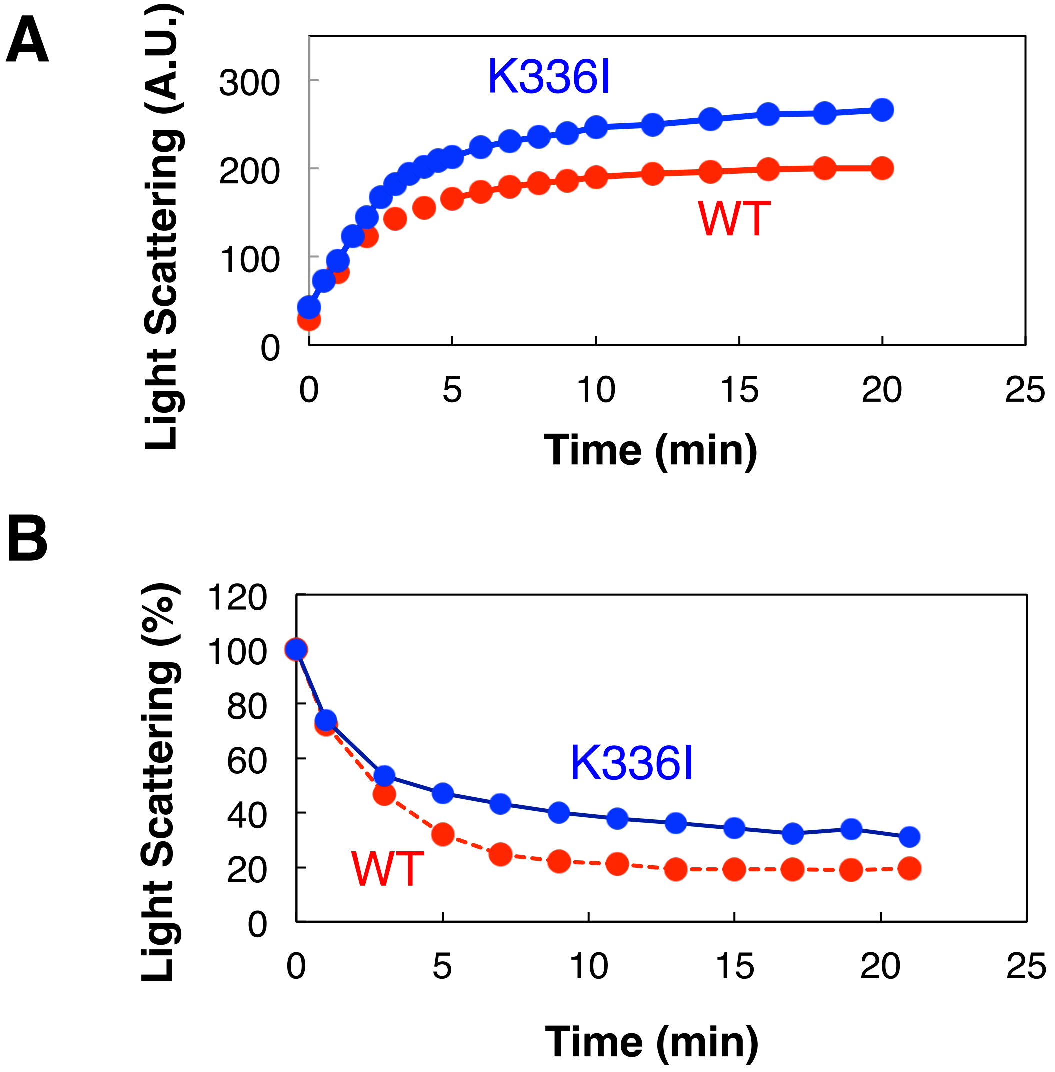

Supplementary Figure S4. Polymerisation and depolymerisation kinetics of K336I and WT actins, monitored by light scattering. (A) Polymerization of K336I actin (10 μM) or WT actin (10 μM) in G-buffer was induced by addition of salt. (B) After polymerisation of K336I actin (5 μM) or WT actin (5 μM), depolymerisation was induced by addition of 36.5 μM Latrunculin A. The plateau levels of K336I actin in the polymerization and depolymerisation were slightly higher than those of WT. We are unable to rule out the possibility that K336I formed nonfilamentous oligomeric structures.

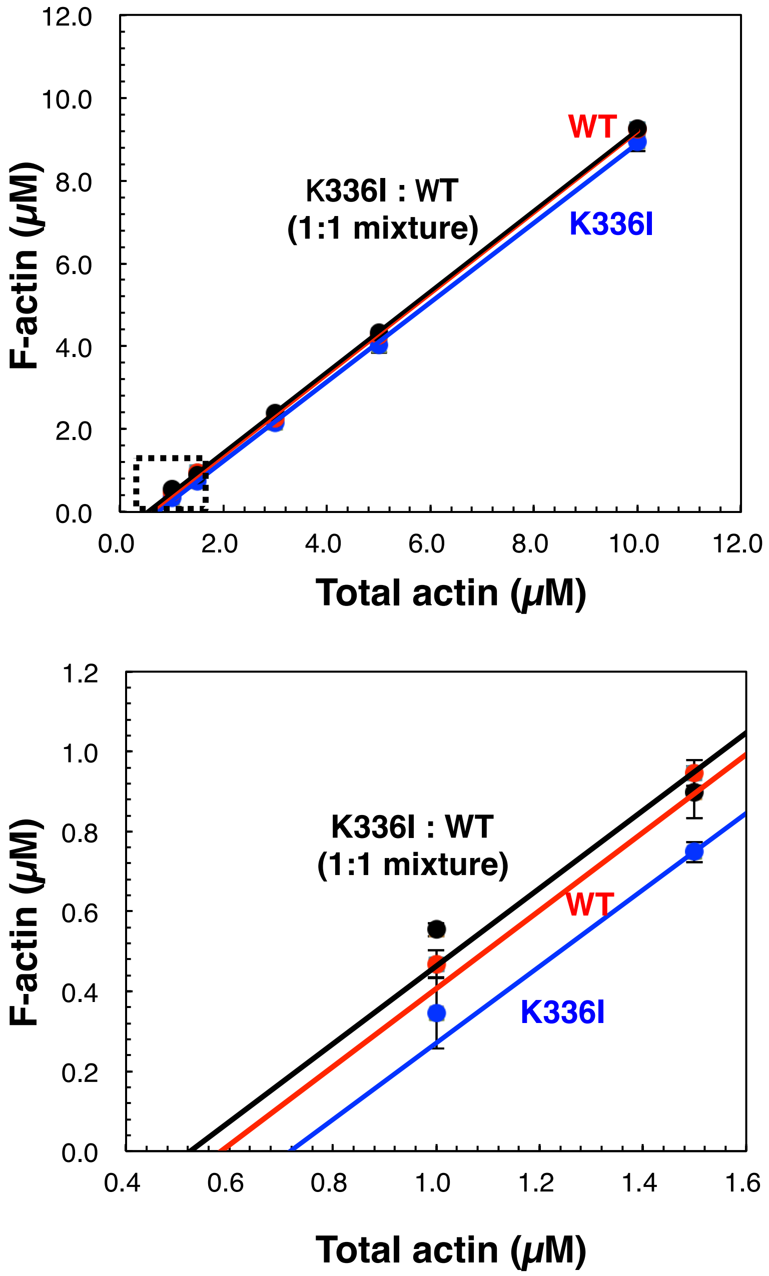

Supplementary Figure S5. Determination of the critical concentration for polymerisation of K336I actin. Plots of [F-actin] versus $[\text{actin}]_{total}$ were fit by the least squares linear regression method, and the intersection of the fitted line with the *x*-axis represents the critical concentration (Cc) for polymerisation of actin. Cc values were as follows: WT = 0.58 μM, K336I/WT = 0.52 μM, and K336I = 0.72 μM. The lower graph is a magnification of the boxed area in the upper graph.

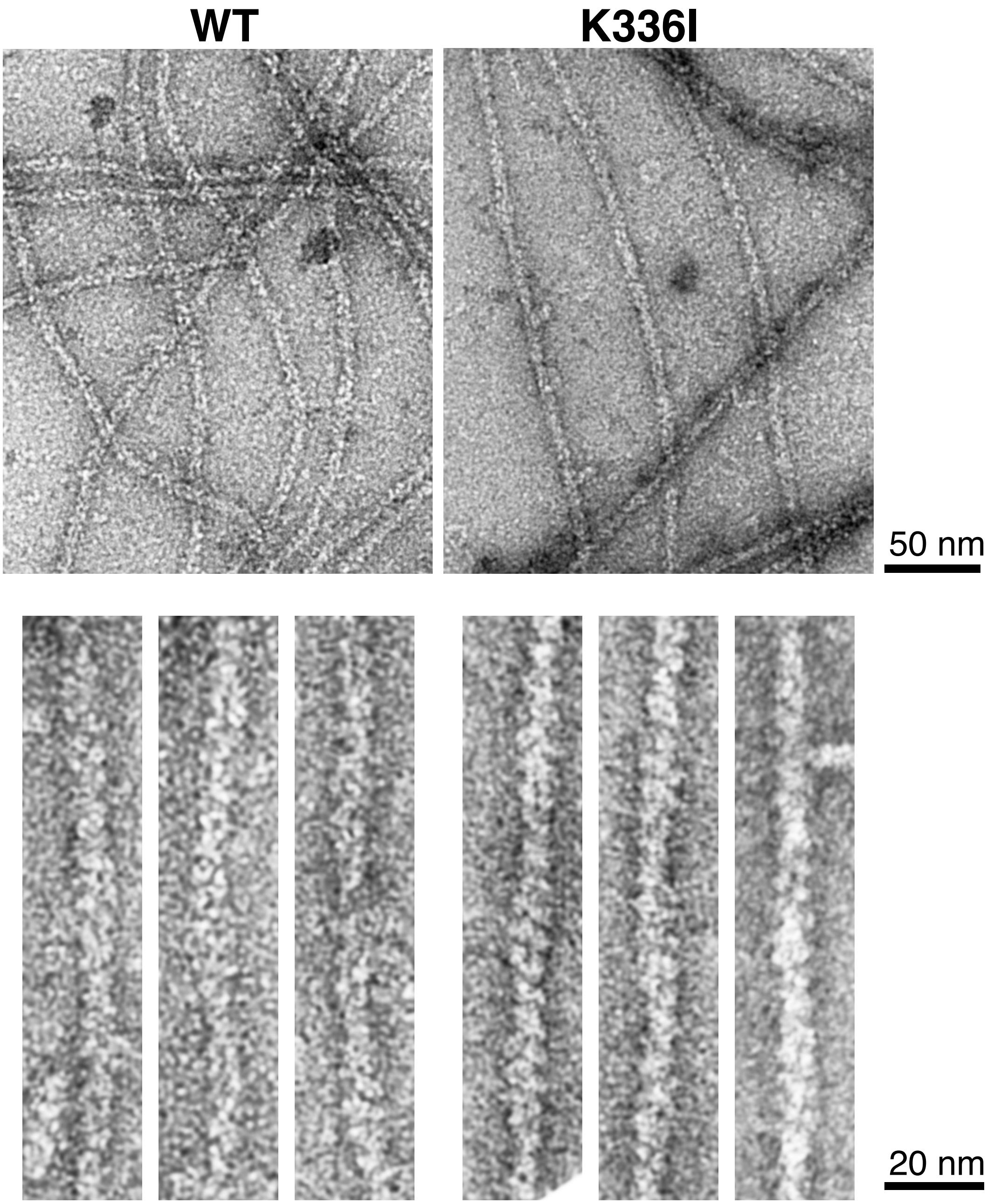

Supplementary Figure S6. Electron microscopy of K336I and WT actin homo-filaments. Filaments were negatively stained with uranyl acetate. The CCD images were Gaussian-filtered to reduce noise. The bottom panels show individual filaments at a higher magnification. There was no discernible difference between K336I and WT filaments.

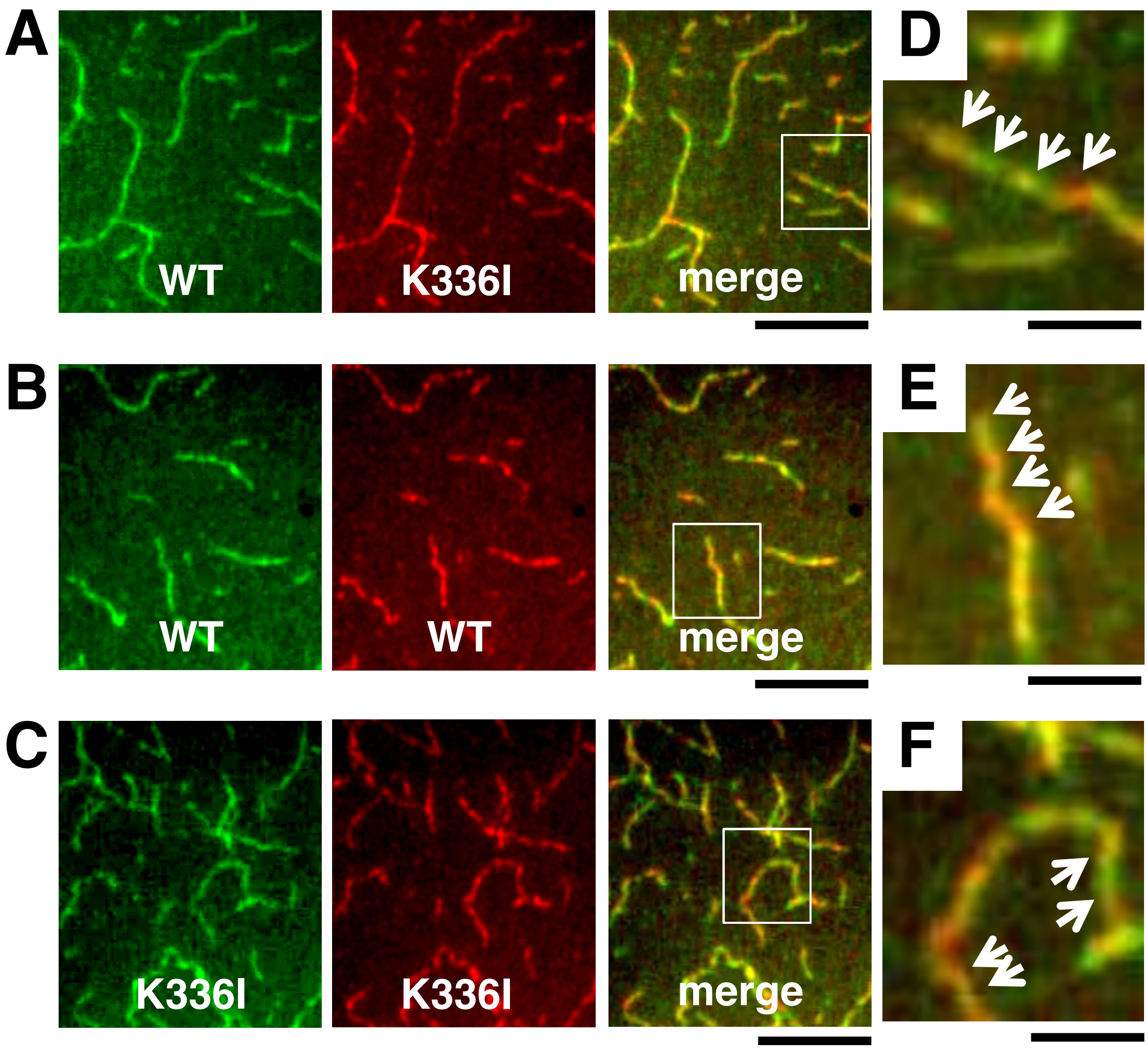

Supplementary Figure S7. Polymerisation of K336I and WT actin observed by fluorescence microscopy. Alexa Fluor–labelled actins were mixed in G-buffer and polymerisation was induced by the addition of salt. (A) Copolymerisation of Alexa Fluor 488–labelled WT actin and Alexa Fluor 594–labelled K336I actin. K336I/WT co-filaments exhibited punctate fluorescence (arrows), but this same pattern was observed both in co-filaments of WT actin (B) and in co-filaments of K336I actin (C) labelled with different dyes, suggesting that it was due to an artefact of labelling with Alexa Fluor dyes, as recently reported [26]. Because of this, it was difficult to determine if K336I and WT actin molecules polymerise homogeneously, or have a weak tendency to segregate from one another in co-filaments. (B) Copolymerisation of Alexa Fluor 488–labelled WT actin with Alexa Fluor 594–labelled WT actin. (C) Copolymerisation of Alexa Fluor 488–labelled K336I actin with Alexa Fluor 594–labelled K336I actin. (D), (E), and (F) are magnified views of the boxed areas in (A), (B), and (C), respectively. Scale bars: 10 μm (A, B, and C) and 3.5 μm (D, E, and F).

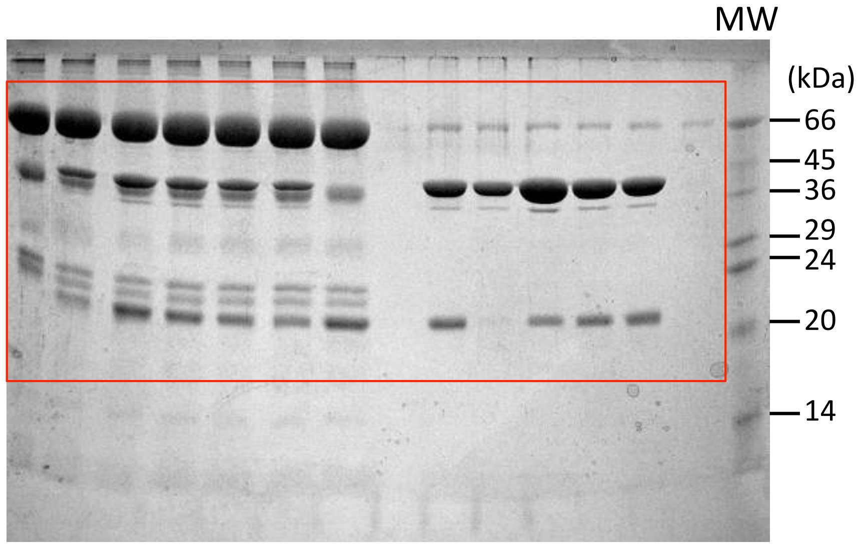

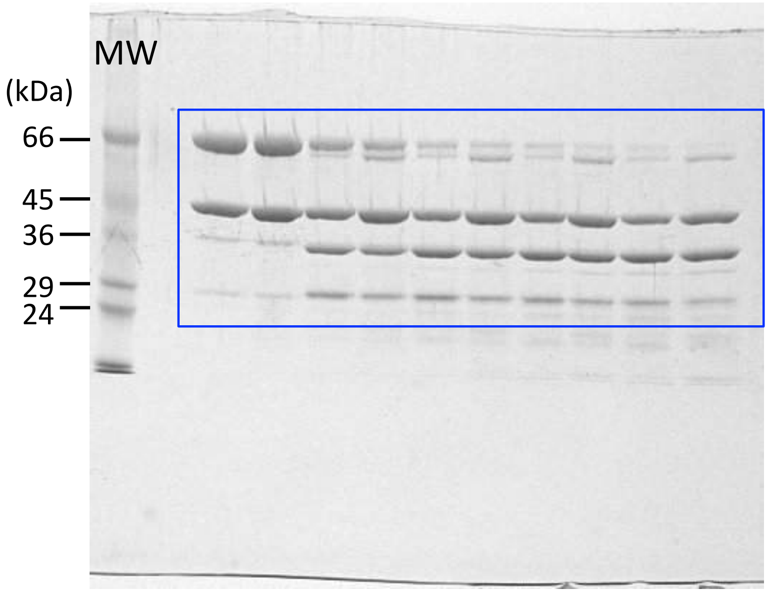

Supplementary Figure S8. Complete SDS-PAGE gel shown in Figure 4A (upper image) and Figure 5B (lower image). The red-boxed area is shown in Figure 4A and the blue-boxed area is shown in Figure 5B. MW, molecular weight markers.

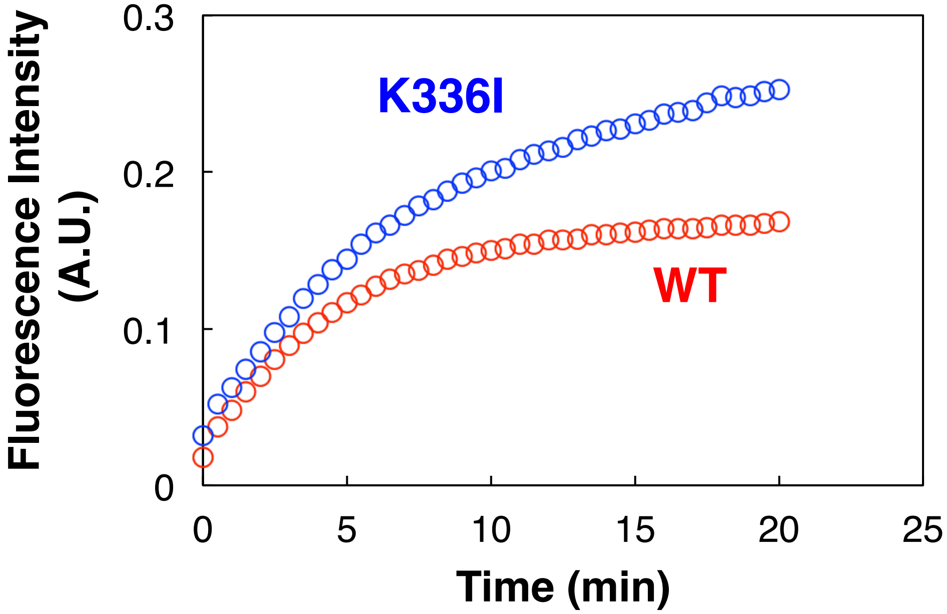

Supplementary Figure S9. Phosphate release from polymerising K336I actin and WT actin. Polymerisation of K336I and WT actin (10 µM) in G-buffer was induced by addition of salt, and Pi-release was monitored using the EnzChek Phosphate Assay Kit.
